# Current challenges in peptide-based drug discovery

**DOI:** 10.3389/fchem.2014.00062

**Published:** 2014-08-08

**Authors:** Laszlo Otvos, John D. Wade

**Affiliations:** ^1^Department of Biology, Temple UniversityPhiladelphia, PA, USA; ^2^Florey Institute of Neuroscience and Mental Health, The University of MelbourneMelbourne, VIC, Australia

**Keywords:** peptide drug development, oral delivery, pharmacokinetics, pharmacodynamics, peptides, grand challenges

The central event of each signaling step in biology is biomolecular recognition. Notwithstanding the importance of nucleic acids, carbohydrates, or lipids in ligand-target interactions, the effectors of most signal transduction processes are peptides. These can be fragments of proteins or stand-alone hormones, cytokines, toxins, antimicrobials, and many other types of peptides. At this point there is no good reason to classify peptides by the number of amino acid residues. We consider peptides as any polyamide (or even biopolymer with ester, thioester, or otherwise modified backbone) that can be made on a contemporary chemical peptide synthesizer. The limit in size is greater than the arbitrary cutoff of 50 amino acids set up by the US Food and Drug Administration (Carton and Strohl, 2013) for proteins and far exceeds that of biological recognition elements. While target recognition can occur with as low as a few residues (Ertl et al., 1991), even wide binding groves can be bound by 30–40 residue long peptides. Thus, in principle synthetic peptides can be used to regulate almost all receptor responses.

The high specificity and low toxicity of peptide drugs derive from their extremely tight binding to their targets. This is due to the large chemical space the side-chain variations of native amino acids cover. Current databases estimate the total number of valid protein-ligand binding sites at 7700 (Khazanov and Carlson, 2013). Calculation based on 17 variable residues (Cys, Met, and Trp are significantly underrepresented in known ligands), show that an 83,000-member tetrapeptide library can be prepared that will essentially cover all unique protein binding regions. As the median length of an active site is 11 amino-acid residues (Khazanov and Carlson, 2013), designed ligands should also be longer. While historically six-residue positional scanning could identify ligands of receptors or epitopes of monoclonal antibodies (Dooley and Houghten, 1993), in our experience receptor agonists are 9–12 residue long (Otvos et al., 2008, 2011a) much like major histocompatibility complex binding peptides (Appella et al., 1995). Antagonists acting on the same receptor binding sites are somewhat shorter (*vide infra*). If it is assumed that conformational preferences improve the binding kinetics but only rarely thermodynamics, then the tremendous specificity of side-chain combinations of peptides over six residues in length can be even further expanded by using non-natural residues. Hundreds of appropriately protected and activated non-natural amino acid derivatives, ready for incorporation into synthetic peptides, are commercially available and indeed are frequently explored in peptide-based drug design. Importantly, chemical biology has provided both backbone and side-chain combinations for exploring an enormous chemical space and is expected to supply peptide chemists with further building blocks suitable for identifying close-to-ideal agonists and antagonists of any biologically important target.

The selectivity of peptide drugs for their target is highlighted by the elevated success rate in clinical trials. According to a biotechnology report (Thomas, 2013), of the 40 approved drugs in 2012, five (12.5%) were peptides compared to 28 small molecule drugs and two monoclonal antibodies (in addition to three enzymes, a cell-based drug and a vaccine). However, in a recent report, the total number of peptide approvals between 2001 and 2012 was 19 (Kaspar and Reichert, 2013). Due to the low number of drug approvals, any particularly successful year can bias the ratios significantly. According to another report, the overall success rate of all drugs entering clinical trials is just 10.4% (Hay et al., 2014). Sixty-five percent of small molecules proceed from Phase I to Phase II in non-oncology applications, a figure identical for peptide/protein drugs. Interestingly peptides/proteins outperform small molecules at the Phase II → Phase III transition stage with 29% for small molecules and 42% for the larger drug candidates. While peptides have traditionally been considered safe in Phase I clinical trials, the public perception is that they are less beneficial in late clinical trials when they are compared side-by-side with different types of treatment modalities. It must be mentioned that peptides are less successful in oncology than in other applications. The cost of large scale peptide production might well-exceed those of small molecule drugs, but if one considers the total cost of the drug development process, the active pharmaceutical ingredient expense will remain under 3% (Otvos, 2014a). In direct opposition to concerns with expensive peptides, the increased clinical success rate, and thus, overall expense/approved drug ratio compared to small molecule chemical entities, make peptide drug development particularly attractive.

The biochemical processes that activated receptors directly or indirectly regulate include protein phosphorylation, nucleic acid transcription, ion transport, and a series of enzyme activities (Yan and Wang, 2012). Ligands (e.g., drugs, hormones, neurotransmitters) bind to receptors and ligand binding activates or inactivates the target and accelerates or inhibits given cellular functions. For peptide-based drug development, a compromise has to be found between the required peptide length and pharmacologically useful levels of receptor activation. The numerous variables include (i) the size and accessibility of ligand binding surfaces, (ii) possible induced fit; (iii) ligand stability and receptor residency time. Contemporary drug development requires nano- or picomolar cellular responses especially for receptor-mediated biological processes. As an example, our leptin receptor agonist peptides must be at least 11 amino acid residues in length (Otvos et al., 2008), similar to other peptide hormone receptor activators (Demchyshyn et al., 2000; Shimizu et al., 2001). For turning peptide agonists into antagonists, the literature data (Hruby, 2002; Sillerud and Larson, 2005) support our own personal experience. Truncation of the sequence or non-natural amino acid replacement usually leads to antagonist or inverse agonist derivatives. Our nine-residue leptin receptor antagonist Allo-aca follows these design rules and shows opposite effects to the agonist both *in vitro* and *in vivo* (Otvos et al., 2011b). Moreover, a library based on the agonist or conformational restriction may allow the selection of peptides with antagonist properties.

In spite of all listed attractive features, ongoing negative public opinion limits more widespread acceptance of peptide drugs. A major challenge in the coming decade will be to modify peptide (polyamide) sequences or properties to overcome these valid concerns and to educate the public to dismiss or reduce the unfounded misconceptions. One of the true drawbacks of peptide drugs is the increased proteolytic instability compared to not only small molecules but also monoclonal antibody therapeutics. The Fc fragment of monoclonal antibodies reshuffle the protein to cells and peptide-Fc conjugates may also be protected from enzymatic cleavage (Boylan et al., 2013). Chemically, both the amide bond and the side-chains can be altered to render the resulting peptidomimetics resistant to proteolytic degradation (Gentilucci et al., 2010). The serum stability assay provides a ready measure of peptide stability and was once considered the most significant secondary screening tool in drug development (Powell et al., 1992). Indeed, serum stability can provide a strong prediction of the all-important pharmacokinetic behavior of drugs.

Nevertheless, the discord between the pharmacokinetics (what the body does to the drug) and the pharmacodynamics (what the drug does to the body) of peptide therapeutics warrants revisiting the importance of stability *per se*. In a mouse model of triple negative breast cancer, Allo-aca is more efficacious than any other current therapy regimen as indicated by survival figures (Otvos et al., 2011c). Due to the similarity of molecular mechanisms in arthritis and cancer, Allo-aca softens rheumatoid arthritis development markers in mice indicating clear and long-term activity of the peptide *in vivo* (Otvos et al., 2011d). At the same time, Allo-aca decomposes within 30 min in human serum and is undetectable in pharmacokinetic measurements 1 h after subcutaneous administration (Otvos et al., 2014). The remarkable *in vivo* efficacies can be explained by the dynamics of Allo-aca binding to the leptin receptor. The estimated binding affinity of biotin-labeled Allo-aca to the ligand binding domain of the receptor is 300 pM and the dissociation rate constant of 1.5 × 10-4/s corresponds to a peptide-receptor complex half-life of nearly 2 h. Allo-aca, and other peptide drugs excel in terms of high activity and target selectivity regardless of poor serum stability and pharmacokinetics. Peptide-based drugs may modify receptor responses significantly longer than standard stability analyses indicate. Indeed, Allo-aca produces weight gain in normal mice even two days after a 0.1 mg/kg bolus subcutaneous administration despite the very short-lived blood levels. In addition, because peptides are rapidly excreted through the kidney, serum stability studies are not representative of true turnover. The 5–10 min T_max_ of peptide drug leads in mouse pharmacokinetics measurements is significantly shorter than true serum presence in humans where the renal clearance rate is 10-fold longer (Sakamoto et al., 1993). Even if they are cleaved into smaller fragments, peptidic metabolites frequently retain the intended biological function (Noto et al., 2008).

Improvement in peptide drug penetration through biological barriers can be achieved by adding modules for passive or active transport (Fasano, 1998). Incorporation of positively charged amino acids, especially at terminal positions improves cell and tissue penetration of peptides (Teesalu et al., 2009; Li and Cho, 2012). Repetitive arginine-containing modules help even nuclear uptake *in vitro* or bioavailability *in vivo* (Wender et al., 2000). One problem is that polycations frequently destroy mammalian membranes as shown by the toxic properties of natural or designer antibacterial peptides that contain large numbers of lysines and arginines (Cudic et al., 2002). Presumably, a safer solution is to conjugate therapeutic peptides to ligands of cell surface receptors. While absorptive-mediated uptake features micromolar saturation constants, generally receptor-mediated uptake is characterized by K_d_ values in the low nanomolar range. Cell surface receptors that can be targeted for internalization of peptides include carbohydrate receptors, lipoprotein receptors, transferring receptors, and receptors involved in cell adhesion. Perhaps the most widely used of these is the incorporation of various sugars to improve tissue penetration, including the transport across the blood-brain barrier. In the first example, coupling a maltose moiety to the N-terminus of a somatostatin octapeptide analog resulted in about 10-fold increased oral bioavailability while maintaining the selectivity and duration of action of the original peptide (Albert et al., 1993). Later this technology was extended to increase in intraintestinal absorption of vasopressin (Kihlberg et al., 1995), the stability of peptide drugs and brain transport of enkephalin analogs (Egleton and Davis, 2005), and more recently to simultaneous intestinal drug absorption and blood-brain barrier penetration of endomorphine-1, an opioid tetrapeptide (Varamini et al., 2012). In our experience, a leptin receptor agonist glycopeptide E1/Aca reduces weight gain in mice fed with high-fat peanut diet in a dose-dependent manner, unlike native leptin protein. In mice undergoing leptin glycopeptide treatment, several obesity-related pathologies (i.e., abnormal metabolic profile and liver histology as well as infertility) are normalized while unglycosylated leptin protein therapy does not show similar positive treatment outcomes (Kovalszky et al., 2010).

Another related area where chemical biology can make critical contributions is increasing the sensitivity of quantitative analysis of peptides. The current limit of quantitation of peptides in plasma by using nano-high-performance liquid chromatography assays is approximately 25 nM (Otvos et al., 2014), around the lower limit of the 10 ng/mL dynamic range (in a 10-mer peptide this is equivalent of 10 nM) in validated pharmaceutical protocols (Zannikos et al., 2000). However, receptor agonist and antagonist peptide drugs act in the pM range, and they are present in mouse blood at a concentration higher than the 100–500 pM IC_50_ value well-beyond the 30–60 min mark that corresponds to the limit of quantitation of the analysis. The situation is less problematic in humans where higher blood volumes are available than in rodents, although the required drug doses in humans are about 12-fold lower than in mice due to differences in the body surface area/weight ratio (Reagan-Shaw et al., 2008). Human serum concentrations of triptorelin, a 10-residue agonist of the gonadotropin-releasing hormone receptor, at 8 ng/mL are already associated with activation of 90% of the receptor population (Romero et al., 2012). In turn, even if the sensitivity of the murine plasma quantification protocol can be increased by a magnitude, later time points will still be missed when highly active peptide drugs are present in the circulation above their IC_50_/EC_50_ figures.

Finally, oral bioavailability (in fact the lack thereof) is a constant discussion topic between peptide drug developers and pharma. Peptides can rarely be absorbed by the intestinal mucosa, and thus cannot serve the appealing lifestyle drug market that includes treatments for weight-loss, smoking, erectile dysfunction, wrinkles, and baldness (Atkinson, 2002). Much effort and monetary resources are expended in the endeavor to make peptides orally active. Chemical and physical modifications that can improve oral bioavailability of peptide drugs include conjugation to passive and active transport enhancers (*vide supra*). In addition to these covalent modification technologies, micro- and nanoparticles further increase peptide delivery options. In most cases, as soon as a peptide drug lead is identified, research is initiated to improve its oral availability. In the latest example, mucoadhesive devices, made of carbopol, pectin and sodium methylcarboxy cellulose, in enteric coated capsules significantly improve the oral bioavailability and pharmacodynamics parameters of an existing peptide drug, salmon calcitonin (Gupta et al., 2013).

However, it must be said that peptide drugs do not necessarily need to be orally available. Many peptide hormones, including insulin, amylin, somatostatin, and human growth hormone are now available in patient-friendly packaging ready for subcutaneous self-administration. The luteinizing hormone-releasing hormone receptor agonist leuprolide, is currently available in a once-a-year implantable device for commercial use. At a research scale, the transdermal delivery of leuprolide can be further improved by using microneedles and/or iontophoresis (Sachdeva et al., 2013). Many peptides can be formulated for intranasal administration, a technique that can utilize olfactory neurons to bypass blood-brain barrier restrictions for central nervous system therapeutics (Charlton et al., 2008). Unsurpassed intranasal efficacies for peptides, comparable to those measured after injection, are also achieved by using alkylsaccharide transmucosal delivery agents (Maggio, 2006). In fact the market is saturated now with personalized, reusable, and virtually painless transdermal or intranasal medical devices for drug administration.

Taken together, the therapeutic potential of peptide-based drugs is increasingly appreciated and their development is both strong and growing rapidly. At this juncture, the role of chemical biology is twofold: solve or improve some of the suboptimal parameters of peptides such as poor pharmacokinetics or lack of oral activity simultaneously with educating biotechnology investors and less informed commercial drug developers about the misconceptions (immunogenicity, high price, unfavorable pharmacodynamics, lack of delivery options) that still linger over peptide-based therapeutics and delay the realization of the use of these highly active and safe therapy options (Otvos, 2014b). By addressing these outstanding issues, peptide-based drugs will finally be accepted as genuine alternatives to traditional small molecule therapeutics.

## Conflict of interest statement

The authors declare that the research was conducted in the absence of any commercial or financial relationships that could be construed as a potential conflict of interest.
